# *EColiCore2*: a reference network model of the central metabolism of *Escherichia coli* and relationships to its genome-scale parent model

**DOI:** 10.1038/srep39647

**Published:** 2017-01-03

**Authors:** Oliver Hädicke, Steffen Klamt

**Affiliations:** 1 Max Planck Institute for Dynamics of Complex Technical Systems, Sandtorstrasse 1, 39106 Magdeburg, Germany

## Abstract

Genome-scale metabolic modeling has become an invaluable tool to analyze properties and capabilities of metabolic networks and has been particularly successful for the model organism *Escherichia coli.* However, for several applications, smaller metabolic (core) models are needed. Using a recently introduced reduction algorithm and the latest *E. coli* genome-scale reconstruction *i*JO1366, we derived *EColiCore2*, a model of the central metabolism of *E. coli. EColiCore2* is a subnetwork of *i*JO1366 and preserves predefined phenotypes including optimal growth on different substrates. The network comprises 486 metabolites and 499 reactions, is accessible for elementary-modes analysis and can, if required, be further compressed to a network with 82 reactions and 54 metabolites having an identical solution space as *EColiCore2*. A systematic comparison of *EColiCore2* with its genome-scale parent model *i*JO1366 reveals that several key properties (flux ranges, reaction essentialities, production envelopes) of the central metabolism are preserved in *EColiCore2* while it neglects redundancies along biosynthetic routes. We also compare calculated metabolic engineering strategies in both models and demonstrate, as a general result, how intervention strategies found in a core model allow the identification of valid strategies in a genome-scale model. Overall, *EColiCore2* holds promise to become a reference model of *E. coli*’s central metabolism.

Constraint-based and stoichiometric modeling has become a standard tool for analyzing key properties and capabilities of metabolic networks and numerous studies have demonstrated the power and wide applicability of this approach^1^,^2^,^3^. An essential prerequisite for a successful application of this modeling framework is the careful reconstruction of the metabolic network model to be investigated. Whereas smaller metabolic models, for instance, of the central metabolism, are often compiled from established biochemical “text book” knowledge, the reconstruction of genome-scale models (covering the entire metabolism of small metabolites) was greatly facilitated due to the availability of sequenced genomes for many organisms^4^,^5^,^6^,^7^. Several tools have been developed to support the reconstruction and gap filling process to eventually obtain high-quality genome-scale models^8^,^9^,^10^,^11^,^12^. It has also become an important task to maintain and continually improve the quality of existing models which is well exemplified by the evolution of genome-scale metabolic models of *Escherichia coli*. The first *E. coli* genome-scale model, *i*JE660^13^, was published in 2000 and already showed a remarkably predictive power. The model was permanently updated and extended leading to model versions *i*JR904^14^, *i*AF1260^15^ and the most recent network *i*JO1366^16^ which is now widely accepted as the reference *E. coli* network reconstruction. Since extensive and reliable data on the biochemistry and genome is available for this model organism, the reconstructed genome-scale models turned out to be exceptionally accurate and proved to be invaluable to gain insights into the metabolism of *E. coli* or to identify intervention strategies for a targeted modification of the metabolism for biotechnological applications^1^,^17^.

However, the increasing size and complexity of genome-scale models have set restrictions on the computational feasibility of some stoichiometric modeling techniques. For example, exhaustive elementary modes analysis^18^ or metabolic flux analysis^19^, can usually not be applied to networks with several thousand metabolites and reactions. Furthermore, basic principles of the operation of metabolic networks, in particular of the central metabolism, can sometimes be more easily studied in smaller core models and the latter are also preferred for kinetic modeling and for educational purposes. For this reason we have recently introduced *NetworkReducer*, a network reduction algorithm that allows the derivation of stoichiometrically consistent submodels from genome-scale models^20^. The derived networks maintain protected parts and phenotypes specified by the user in a preprocessing step and guarantee that quality features of the genome-scale reconstruction such as mass and charge balance are preserved. Whereas the general applicability of *NetworkReducer* has been demonstrated before, the goal of the current contribution is to derive a widely applicable core model of the central metabolism of *E. coli* from a reference genome-scale network and to analyze its properties in relation to the genome-scale parent model.

Several different small-scale models of the central metabolism of *E. coli* have been used in the literature. However, almost all of them were derived in a bottom-up manner and are therefore not related to and consistent with a corresponding genome-scale model. One exception is the *E. coli* core model presented by Orth *et al*.^21^ which is one of the most frequently used models of this kind. The model, in the following denoted by *EColiCore1*, was manually derived from the genome-scale model *i*AF1260 for educational purposes. Whereas *EColiCore1* perfectly fits the demands it was developed for, the stoichiometric properties are closely related but not fully consistent with its genome-scale parent model *i*AF1260^20^. When we introduced *NetworkReducer*^20^, we showed how one can derive a fully consistent stoichiometric core network from *i*AF1260 having the same scope as *EColiCore1*. However, even this core model has several drawbacks. First of all, it was not derived from the latest reference genome-scale model *i*JO1366. Secondly, it lacks some central metabolic pathways such as the Entner-Doudoroff and the methylglyoxal pathway. Furthermore, we wanted to protect a larger set of phenotypes in the core model including feasibility of growth on a selection of standard substrates as well as producibility of standard (fermentation) products by *E. coli*. One major goal of the present contribution was therefore to derive a stoichiometric model of the central metabolism of *E. coli* from *i*JO1366 that fulfills these requirements and therefore holds the potential to become a reference model of the central metabolism of *E. coli* accessible for all types of constrained-based modeling. Another central goal of this study is to systematically compare key properties of the derived core model, *EColiCore2*, with those from its genome-scale parent model *i*JO1366. This analysis, which is the first of this kind, includes a comparison of the solution space with respect to flux ranges, reaction essentialities, and production capabilities. We demonstrate that *EColiCore2* is capable to describe several key properties of *i*JO1366 including those that were not explicitly accounted for. Generally, as intended, the derived core model reflects the degrees of freedom in the central metabolism of *E. coli* very well while neglecting redundancies along biosynthetic routes. A full enumeration of the complete set of elementary modes or of metabolic intervention strategies becomes possible in *EColiCore2.* Using ethanol as a relevant product, we also compare metabolic engineering intervention strategies in the core and the genome-scale model and demonstrate how the core model can even be used as a basis for the identification of metabolic engineering strategies with higher cardinalities in the genome-scale model that were computationally not tractable before.

## Methods

### Network reduction

For deriving the *E. coli* core model we used the recently introduced network reduction algorithm *NetworkReducer* which takes a given large-scale metabolic network as input and derives a smaller subnetwork while keeping desired features of the full network intact^20^. Among others, properties to be preserved may comprise sets of protected metabolites and reactions that must not be deleted. In addition, protected functions and phenotypes can be specified that have to be maintained in the reduced network. Each protected functionality *k* is described by a corresponding set of linear inequalities:


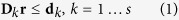


with *s* denoting the number of protected functions and phenotypes. In a first (pruning) step, *NetworkReducer* iteratively removes non-protected reactions and metabolites while checking that none of the protected functions are violated. Afterwards, an optional network compression step can be applied which, for example, lumps reaction sequences (unbranched pathways) into single reactions. This second operation further reduces the size of the network but does not change the space of feasible steady-state flux distributions. For further details on *NetworkReducer* we refer to the original work^20^.

### Genome-scale *E. coli* model

The latest reconstruction *i*JO1366 was used as the gold standard for a well-curated genome-scale stoichiometric network model for *E. coli*’s metabolism. We introduced some minor modifications to *i*JO1366 prior to the application of the network reduction routine:

1. We added external metabolites for all reactions where compounds are secreted into or taken up from the environment. This allows a more convenient consideration of mass balances of those metabolites (especially in the compressed network) but does not change the solution space.
2. Further, we allowed reaction R_nadh16tpp (NADH dehydrogenase) to be reversible as otherwise succinate excretion is obligatory under anaerobic conditions (which would be in conflict with experimental findings^22^). Experimental evidence for the reversibility of the NADH dehydrogenase reaction has been reported^23^.
3. When applying the *NetworkReducer* routine, we found that the malate synthase reaction and formate (or hydrogen) excretion became essential reactions because they are needed to consume formate and glyoxylate formed as byproducts of some synthesis pathways for biomass precursors. This behavior would not reflect reality. In fact, in the genome-scale model, there are different routes that consume these metabolites (hence, the aforementioned reactions are not essential in *i*JO1366), however, these pathways would be lost in the reduction process. To avoid such an undesired behavior of the reduced model, we demanded that growth without formate excretion and malate synthase is possible and replaced metabolites M_glx_c (glyoxylate) and M_for_c (formate) in the reactions R_GLYCTO4, R_GLXCL, R_AMPMS2, R_DB4PS, R_FTHFLi, R_GTPCI and R_GTPCII2 by auxiliary metabolites M_glxBIO_c and B_forBIO_c, respectively. These changes reflect the assumption that consumption of glyoxylate and formate produced during biomass synthesis is realized via these reactions. Importantly, this modification does not produce new steady-state flux distributions, it only excludes those solutions that would render malate synthase and formate (or hydrogen) production essential.

For clarity, the slightly modified *i*JO1366 model will be denoted by ECGS (*E. coli* genome-scale) throughout the manuscript. Flux bounds were set as in the original model *i*JO1366, in particular, a maximal substrate uptake rate of 10 mmol/gDW/h was used (if not stated otherwise) and the ATP maintenance demand was set to 3.15 mmol/gDW/h. The flux range of all reactions with unknown capacities were set to [−1000, +1000] for reversible and [0, +1000] for irreversible reactions.

### Protected parts and phenotypes

**Figure 1:**
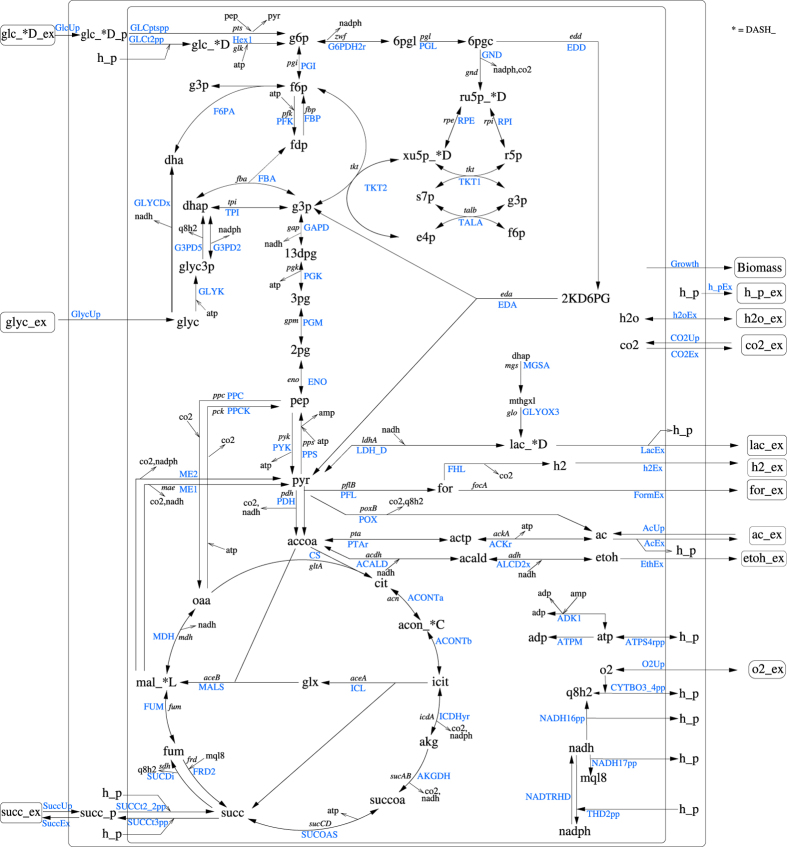
Graphical representation of the compressed *E. coli* core network ECC2comp. Reaction names are given in blue. Except for the external metabolites, the (compressed) exchange reactions, and the renamed biomass synthesis reactions (here indicated as “growth”), all metabolites and reactions are also contained in *i*JO1366 and ECGS and are also part of the uncompressed core model ECC2 (ECC2 contains additionally biosynthesis pathways that are compressed to one ”growth” reaction in ECC2comp).

Next we specified the protected parts and phenotypes of the genome-scale model to be retained by *NetworkReducer*. As the goal is to construct a core model that represents the central metabolism of *E. coli* in detail while neglecting redundancies in anabolic synthesis pathways we protected the reactions and metabolites involved in glycolysis, pentose phosphate pathway, Entner-Doudoroff pathway, tricarboxylic acid cycle, methylglyoxal pathway and key reactions of the electron transport chain. In addition, six major anaplerotic reactions (including the glyoxylate shunt) and two NAD(P) transhydrogenase reactions were protected. To account for biomass synthesis we demanded that the reaction R_Ec_biomass_iJO1366_core_53p95M of ECGS must be maintained. In total, 65 reactions and their reactants have been protected in the central metabolism as listed in the Supplementary Information (see also Fig. 1).

The set of protected reactions does not yet guarantee that the reduced network is able to represent any specific functionality. For example, growth would not be possible with the defined set of protected reactions as almost all reactions needed for synthesis of biomass components (specified in the biomass synthesis reaction) are not included in this set. Here we exploit the possibility of *NetworkReducer* to protect not only parts but also behaviors or phenotypes. We therefore demand that the reduced network must allow for growth on four representative carbon substrates (glucose, glycerol, acetate, succinate) with at least 98% of the maximal growth rate achievable with the ECGS model (Table 1). This ensures that the reduced network contains at least all protected reactions and additionally a set of biosynthesis routes that produce all components consumed by the biomass synthesis reaction. Likewise, optimal growth on glucose under anaerobic conditions was also protected. Furthermore, we demanded that the production of most important fermentation products (ethanol, lactate, succinate, acetate, formate, and hydrogen) is possible. In total we protected eleven different functionalities as summarized in Table 1 (a full description of the protected parts and phenotypes can be found in the Supplementary Information). For all calculations, we considered growth on minimal medium with the chosen substrate. Hence, uptake of other carbon metabolites was prevented by setting the corresponding reversibility constraint of exchange reactions to irreversible (only export allowed) in ECGS.

The network reduction and all other calculations were done with *CellNetAnalyzer*^24^. The input genome-scale network (ECGS), the pruned (*EColiCore2*) and the compressed pruned (*EColiCore2*^*comp*^) networks are provided in SBML format in Supplementary Dataset Files. Additionally, these three network models are also available as *CellNetAnalyzer* projects on the repository website of *CellNetAnalyzer* (http://www2.mpi-magdeburg.mpg.de/projects/cna/repository.html).

## Results

### Basic properties of *EColiCore2* (ECC2)

After applying *NetworkReducer* to the *i*JO1366-based genome-scale model ECGS as described in the Methods section (only pruning, no compression), we obtained *EColiCore2* (in the following abbreviated by ECC2), a core model of *E. coli*’s metabolism where all the specified protected parts and phenotypes have been maintained. ECC2 contains 499 reactions (thereof 198 reversible) and 486 internal as well as 33 external metabolites (Table 2). Only 65 of the 499 reactions were explicitly protected during reduction. 399 additional reactions were kept by the algorithm to enable biomass synthesis and another 35 reactions were retained to allow for the transport of metabolites to and from the extracellular environment. Despite the still relatively high number of reactions and metabolites only 25 degrees of freedom remained (compared to 817 in the original ECGS model), making this network accessible for detailed studies including elementary-modes analysis (see below).

We emphasize that network ECC2 is not the only possible solution and other subnetworks can solve the reduction problem equally well. However, by definition, all alternative networks share the set of protected parts (metabolites and reactions) and protected phenotypes. Further, by altering the order of removed reactions, we found that differences in alternative reduced networks occur mainly in alternative routes for the synthesis of biomass precursors. Our focus was to obtain a core model that (a) represents *E. coli*’s central metabolism in detail and (b) preserves major metabolic phenotypes. Therefore, we consider ECC2 as a qualified solution suitable for further in-depth analysis.

The demanded biomass yields for the different substrates are achieved by ECC2. For all substrates, the maximal biomass yields in ECC2 are even above 99.9% of the genome-scale maximum except for aerobic growth on acetate where 98.8% of the maximal biomass yield of ECGS is achieved (Table 1). Regarding the standard fermentation products, although only the *possibility* of anaerobic production of lactate, succinate and ethanol was demanded, their maximal production rates correspond exactly to those of ECGS (in those cases, the protected reactions already guarantee these maximal rates). The maximal product for formate, hydrogen, and acetate in ECC2 compare also well with what is known (and expected) from experimental observations, however, they are much higher in ECGS (Table 1). For example, the maximal molar acetate yield in *i*JO1366 for aerobic growth on glucose is 2.93 (but only 2.0 in ECC2). We refrained from demanding that ECC2 reproduces these extremely high product yields as they are not expected to be of relevance under physiological conditions (see also discussion below).

### Compressed *EColiCore2* (ECC2comp)

Applying also the optional compression step of *NetworkReducer* to ECC2 generates an even more reduced network, ECC2comp, where coupled reactions (e.g., from linear reaction sequences) are lumped into overall reactions. The lumped reactions can be classified into two different categories. The vast majority (399) are reactions that are fully coupled (form an enzyme subset) with the biomass synthesis reaction. In addition, 21 reactions of the ECC2 model involved in chains of transport reactions (secreting metabolites from the cytosol via periplasm to the extracellular compartment) have been lumped as well. The compression thus further reduced the number of reactions and metabolites down to 79 reactions and 61 internal and 33 external metabolites. Initially, ECC2comp contained 25 degrees of freedom as ECC2, however, for better handling, we split up the reversible exchange reactions for carbon dioxide (CO_2_), acetate, and succinate into two irreversible (uptake and excretion) reactions respectively thereby increasing the number of reactions to 82 and the degrees of freedom to 28 (Table 2). Furthermore, ECC2comp contained seven conservation relations (e.g., AMP+ADP+ATP+Pi = const.) which were eliminated by defining one metabolite of each conservation relation as external (e.g., Pi in the above example). This does not change the solution space of ECC2comp but avoids potential numerical problems. ECC2comp has a final dimension of 82 reactions and 54 internal metabolites (Table 2) and is depicted in Fig. 1.

Due to the lumping of biosynthetic reaction chains, the biomass synthesis reaction of the compressed network ECC2comp includes different stoichiometric coefficients and also different precursors (e.g., pyruvate or oxaloacetate instead of amino acids) and biomass byproducts compared to ECC2 and ECGS. However, the adjusted stoichiometric coefficients and the new precursors reflect the metabolites and their amount needed to produce all the building blocks contained in the original biomass synthesis reaction of ECC2 and ECGS (see also ref. 20). ECC2comp is therefore fully consistent with the ECC2 model and preserves all protected parts and phenotypes (Table 1). In particular, all feasible steady-state flux distributions of ECC2comp can be unambiguously mapped to flux distributions of ECC2 (and vice versa). Likewise, the elementary modes (EM) of ECC2comp and ECC2 are equivalent. For example, whenever the growth reaction is contained in an EM of ECC2comp, the growth reaction and the 399 reactions that are strictly coupled with the growth reaction are contained in the corresponding EM of ECC2. Since the compression step does not change the properties and capabilities of the network we will concentrate the following analyses on network ECC2. In many realistic applications it will, however, be sufficient and more efficient (e.g., regarding memory requirements; see below) to use the compressed model ECC2comp instead of ECC2.

### Comparison of key properties in the genome-scale and in the core model

To compare key properties of the reduced core network ECC2 with those from its genome-scale parent model ECGS we calculated several characteristics in both models including flux ranges, reaction essentialities, and metabolite producibilities. The feasible flux spaces are also compared by means of analyses of production envelopes. Finally, using a case study for ethanol production, the value of ECC2 for the identification of metabolic engineering strategies, in the core ECC2 *and* in the genome-scale ECGS model, will be demonstrated.

### Flux ranges

Flux ranges were determined in ECC2 and ECGS for optimal growth of *E. coli* with glucose as sole substrate (for flux bounds see Methods). The growth rate was fixed to its near optimal value (0.98 h^−1^) and for all remaining reactions the minimal and maximal flux values were calculated for both networks. With ECC2 being a subnetwork of ECGS, it follows that the flux range of a reaction in ECC2 will be contained in the flux range of this reaction in ECGS. For 290 out of the 499 reactions in ECC2 (58.1%) a (close to) perfect agreement with the corresponding flux ranges in ECGS can be observed (difference less than 0.1 mmol/gDW/h) and for additional 153 reactions (30.7%) a good agreement can be found (flux range differs less than 1 mmol/gDW/h which is less than 10% of the maximal substrate uptake rate) (Table 3). For only 11.2% of the reactions larger discrepancies in the flux ranges can be found; 5.8% of the flux ranges differ up to 10 and 5.4% by more than 10 mmol/gDW/h. Many of these reactions are related to transport of extracellular metabolites that can be cycled between the different compartments in ECGS but not in ECC2. For example, the flux range for the exchange flux of the metabolite Fe2_p is broader than 1000 (r_min = −1000, r_max = 0.23) in network ECGS but zero in ECC2 as its value is fixed to −0.008 (exactly the amount needed to synthesize the biomass for the fixed (optimal) growth rate). Further, even some reactions of the central metabolism (e.g., the phosphate acetyltransferase (R_PTAr) and succinyl-CoA synthase (R_SUCOAS)) reach their minimal (−1000) or/and maximal flux bound (+1000) in ECGS indicating that these fluxes are unlimited due to internal cycles. Since these cycles do not exist anymore in ECC2 the flux ranges of these reactions are much smaller in the core model.

### Reaction essentialities

Reaction essentialities for growth represent another important functional network property. We focused on reaction essentialities for growth on glucose. Separately for each reaction we fixed the rate to zero and maximized the growth rate. A reaction knockout is considered to be without significant effect if the maximal possible growth rate (μ_max_) of the mutant is still above 95% of the wild type μ_max_. If μ_max_ is reduced by more than 5% but growth still possible with more than 5% of the wild type μ_max_ then the reaction is considered growth-limiting but not essential. Finally, if μ_max_ falls below 5% of the maximal growth rate in the wild type, the reaction is considered to be lethal.

Before comparing the essentialities, it is important to notice that all reactions being essential in network ECGS will be essential in network ECC2, too, because ECC2 is a subnetwork of ECGS and, therefore, the solution space of ECC2 a subset of the feasible solution space of ECGS. We found that 411 out of the 499 reactions in ECC2 are essential whereas only 290 of them are also essential in ECGS (Table 3). The 121 reactions being essential in ECC2 but not in ECGS have no effect if knocked out in ECGS. The classification of reactions being growth-limiting but not essential is identical in both networks except for one reaction (pyruvate dehydrogenase, R_PDH) for which the classification “no effect” in ECGS switched to “medium effects” in ECC2 (with knockout of R_PDH the maximal growth rate dropped to 94% in ECC2 compared to 99% for ECGS). Consequently, knockout of 193 of the 499 reactions contained in ECC2 are not growth-limiting in ECGS but only 71 of them do not suppress growth in ECC2. The observed discrepancy regarding essential reactions is not surprising and was expected as many parallel pathways for synthesis of certain biomass components exist in ECGS (making many reactions dispensable) but were removed in ECC2 (through which all reactions along biosynthesis routes become essential). However, for the reactions in the central metabolism (that were protected during the reduction process) the concordance in predictions of reaction essentialities in ECC2 an ECGS is almost perfect (Table 3). Only two deviations can be observed: the adenylate kinase (R_ADK1) is predicted to be essential in ECC2 but not in network ECGS. Due to the deletion of alternative recycling routes for AMP, R_ADK1 is the only available reaction in ECC2 that consumes AMP produced by other reactions. The other deviation corresponds to R_PDH that was already described above. Hence, as a desired feature, the original essentialities of the reactions in the central metabolism are very well reflected by the ECC2 model.

### Producibility of metabolites

For growth on glucose, we determined the maximal yield achievable for each metabolite in ECGS and ECC2. The glucose uptake rate was set to unity for both networks (ATP maintenance demand and uptake of other substrates was set to zero for these calculations). For each internal metabolite a corresponding excretion reaction was temporarily introduced and then maximized.

Similar as for reaction essentialities, the maximal yield of any metabolite in ECC2 can never be greater than in ECGS since all feasible flux distributions in ECC2 are also possible in ECGS. For the vast majority of metabolites (77.8%, Table 3) we found that the corresponding yields agree (almost) perfectly (at least 95% of the maximal yields of those metabolites in ECGS are also achieved in network ECC2). For 11.3% of the metabolites, ECC2 enables yields that are below 95% but still above 80% of the respective maximal possible yield in ECGS. Only 10.9% of the metabolites have optimal yields that are reduced by more than 20% in ECC2. As in the case of reaction essentialities most of the differences in maximal yields are related to metabolites that are synthesized along biosynthetic routes and are required in very small amounts only in the biomass synthesis reaction. However, unexpectedly, six metabolites from the central metabolism (all involved in protected reactions) showed larger deviations: ac_c (acetate), actp_c (acetylphosphate), akg_c (oxoglutarate), for_c (formate), glx_c (glyoxylate) and h2_c (hydrogen). Our analyses revealed that these deviations are rather due to artificial properties of ECGS than to lost desired properties of ECC2. For example, the maximal yields of akg_c and glx_c are as high as 1.29 and 3.34 in ECGS but only 1.0 and 2.0 in ECC2. The high values in ECGS can only be achieved if net CO_2_ fixation would be conducted by *E. coli* which is unlikely as such a behavior has, to our knowledge, not been reported yet for these metabolites.

### Production envelopes

Production envelopes (PEs) are projections of the solution space of feasible steady-state flux distributions on a two-dimensional plane. The growth rate is typically projected on the x-axis and a selected product synthesis (excretion) rate on the y-axis. PEs can be computed by means of sequential FVA where the growth rate is incremented in discrete steps from its minimum to its maximum and the minimal and maximal product synthesis rate is computed for each step. It should be noted again that, since ECC2 is a sub-network of ECGS, any PE of ECC2 will be contained in the corresponding PE of ECGS.

**Figure 2:**
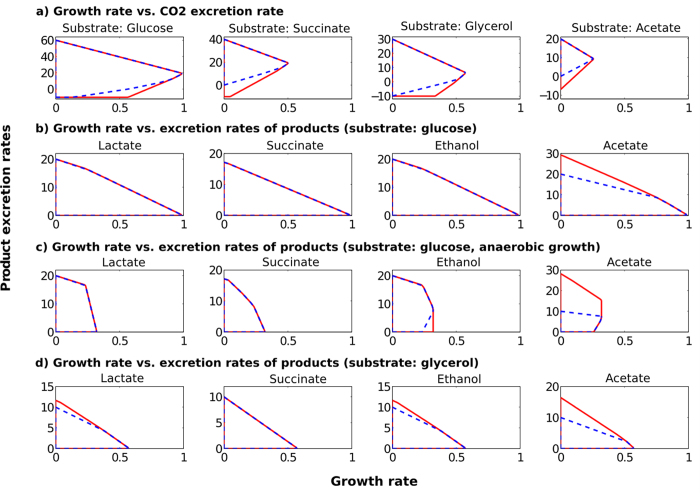
Production envelopes for different substrates and environmental scenarios. The growth rate (x-axis) is given in h^−1^ and the product excretion rates (y-axis) in mmol/gDW/h. For each production envelope, the respective maximal substrate uptake rate was set to 10 mmol/gDW/h and the non-growth associated ATP maintenance demand to 3.15 mmol/gDW/h (see also Methods). Red lines: production envelopes for ECGS; blue dashed lines: production envelopes for ECC2.

A selection of representative PEs is given in Fig. 2 (the substrate uptake rate was set to 10 mmol/gDW/h for all substrates; for other flux constraints see Methods). The top row shows the PEs for all substrates with respect to growth rate versus CO_2_ release under aerobic conditions. It can be seen that the maximal growth and CO_2_ production rates are well represented by the reduced model while some deviations occur for the lower boundaries of CO_2_ release. For example, while the genome-scale model ECGS allows for CO_2_ fixation with acetate and succinate as substrates this behavior is not reflected by ECC2. The second and third row show the PEs for growth versus synthesis rates of typical fermentation products with glucose as substrate under any and under exclusively anaerobic conditions, respectively. Except for acetate, the PEs are either identical or, for the case of ethanol production under anaerobic conditions, show a large overlap confirming that the maximal yields of those products in ECGS are also reachable by ECC2. As was already mentioned above, whether the predicted maximal acetate yield with ECGS of almost 3 mol acetate per mole glucose is a realistic value remains questionable. In particular, homoacetic acid fermentation in *E. coli* (without incorporation of heterologous pathways) as predicted by ECGS has, to our knowledge, not been observed so far. Therefore, we did not protect this property in ECC2 and consider this deviation rather as correction than as restriction. The last row in Fig. 2 shows the PEs with glycerol as substrate. As for glucose, the PEs of ECC2 and ECGS overlap largely except for maximal acetate production.

### Elementary modes

As one goal of the whole reduction process, with the reduced degrees of freedom in ECC2 and ECC2comp, these models become accessible for the enumeration and subsequent analysis of elementary modes (EM). In the following we will discuss the EM of ECC2 keeping in mind that, as already mentioned, the EM are equivalent for both networks (up to a reaction mapping for all lumped reactions of ECC2comp to the corresponding involved reactions in ECC2) and storage of EM from ECC2comp will require less than 20% of the memory needed to store the EM of ECC2 (82 vs. 499 reactions). Table 4 summarizes the numbers of EM for growth on each of the four considered substrates (for glucose we also calculated the subset of EM for anaerobic growth). The highest number (about 3.26 million) of EM was computed for growth on glucose. Although this is still a large set of EM describing a multitude of glucose conversions by the central metabolic pathways, the calculation could be finished on a standard desktop computer in less than 5 minutes and a convenient handling of these EM is possible. In comparison, partially much less EM were observed for the other substrates and for exclusively anaerobic growth on glucose.

A comparison of the set of EM from ECC2 and ECGS is not possible. So far, the computation of EM for genome-scale models is limited to approaches that compute the shortest EM^25^ or sample a subset of EM via heuristic approaches^26^. As a very important and useful property, all EM computed in ECC2 are also valid EM in ECGS because ECC2 is a subnetwork of ECGS. The EM of the reduced ECC2 model thus provide a representative set of EM of the genome-scale ECGS model with respect to the protected parts and phenotypes.

### Comparison of metabolic intervention strategies for high-yield production of ethanol

In the following we want to study how metabolic engineering strategies identified in the ECC2 model relate to the corresponding intervention strategies computed in ECGS. As a relevant product we chose ethanol which has previously been used as a “benchmark” case in other studies^27^,^28^. Different computational methods for calculating metabolic engineering strategies from stoichiometric models have been described in the literature^9^,^29^,^30^,^31^. Here we make use of the (constrained) minimal cut sets (MCS) approach which, among other applications, can be used for enumerating knockout strategies that couple growth with product synthesis (the product becomes a mandatory byproduct of biomass synthesis). Originally, MCS were calculated from the network’s EM^27^,^28^, but recently an algorithm has been presented which allows the enumeration of smallest MCS also in genome-scale networks where EM are usually not calculable^28^.

Here we consider production of ethanol from glucose under anaerobic conditions (oxygen uptake rate set to zero). The maximal glucose uptake rate was increased to 18.5 mmol/gDW/h to reflect the higher glucose conversion rates under anaerobic conditions. We excluded pseudo (transport and excretion) reactions from the list of possible knockout targets leaving only reactions that can be targeted by genetic modifications as knockout candidates. We designed the MCS problem as follows: the engineering goal with respect to product formation was formulated by demanding a minimal product yield of 1.4 mol ethanol per mol glucose consumed while growth should be feasible with a minimal rate of 0.05 h^−1^.

For these constraints, we calculated the complete set of MCS with up to ten reaction deletions for both models, ECGS and ECC2. In total, we found 2176 MCS in ECC2 and 44066 MCS in the genome-scale network ECGS (Table 5). Each MCS represents one suitable knockout strategy to achieve the desired phenotype in the respective network. Table 5 shows the size distribution of the MCS in both networks indicating that at least 5 reaction knockouts are required in ECC2 whereas a minimum of 6 reaction deletions are needed in ECGS. The MCS from ECC2 are composed of combinations from 38 different reactions. In ECGS, 169 different reactions are contained in the MCS, of which 72 are part of ECC2. On the other hand, there exist five reactions (glycerol-3-phosphate dehydrogenase (R_G3PD5), malic enzyme (R_ME1), NADH transhydrogenase (R_NADTRHD), pyruvate dehydrogenase (R_PDH), pyruvate oxidase (R_POX)) that were knockout candidates in some MCS of ECC2 but no targets in any MCS of network ECGS (however, these reactions are certainly involved in MCS with higher cardinalities not calculated herein; see below). From the 39 reactions of ECC2 that were candidates in ECGS but not in ECC2, 32 are essential for biomass synthesis and therefore not deletable in ECC2. The remaining seven reactions are acetaldehyde dehydrogenase (R_ACALD), ATP synthase (R_ATPS4rpp), 2-dehydro-3-deoxy-phosphogluconate aldolase (R_EDA), 6-phosphogluconate dehydratase (R_EDD), fructose 6-phosphate aldolase (R_F6PA), fructose-bisphosphatase (R_FBP) and periplasmatic NAD(P) transhydrogenase (R_THD2pp). Finally, 33 reactions are detected as knockout candidates for both networks, ECC2 and ECGS.

### Relationships between MCS from ECC2 and ECGS

**Figure 3:**
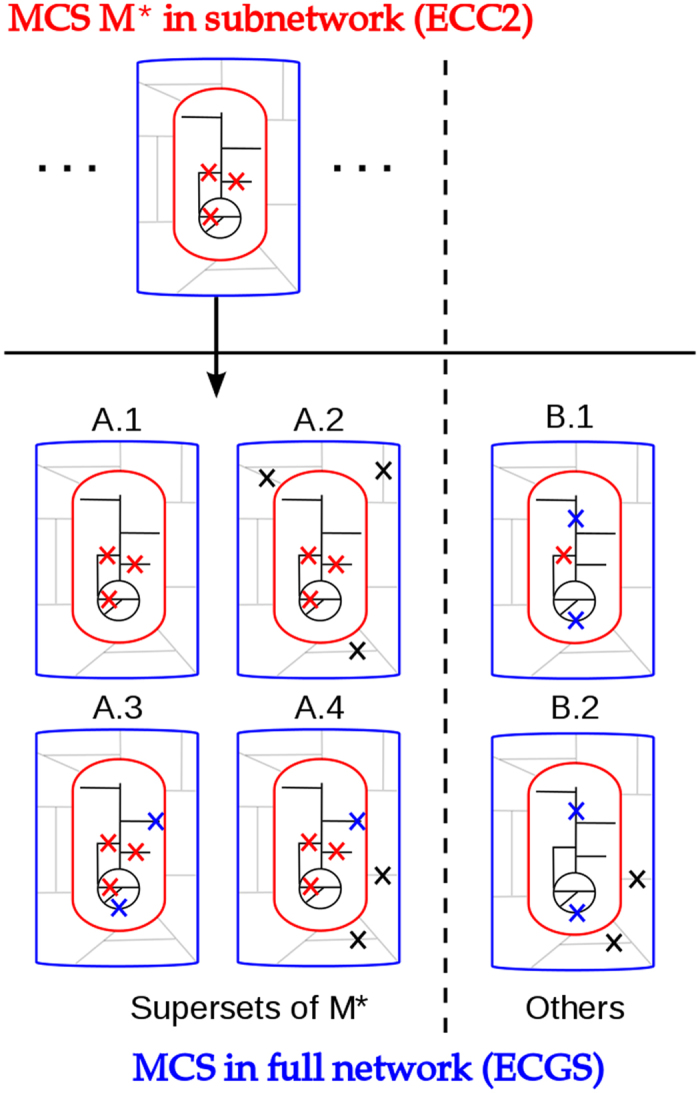
Categories of minimal cut sets (MCS) in ECGS with respect to a given (specific) MCS M* of ECC2. The red oval comprises ECC2 and its corresponding reactions (black lines). The blue box indicates the ECGS model (including ECC2 and additional reactions). Grey lines: reactions of ECGS that are not in ECC2. Red crosses: reaction deletions contained in M*. Blue and black crosses indicate (additional) knockouts in an MCS of ECGS not contained in M*. Blue crosses: (additional) deletions of reactions in ECGS which are contained in the ECC2 part (but not contained as cuts in M*). Black crosses: (additional) deletions of reactions in ECGS outside the ECC2 part.

The property of ECC2 to be a subnetwork of ECGS again helps us to derive important relationships between the MCS in ECC2 and the MCS in ECGS. In the following, an MCS of ECC2 is denoted by M_ECC2_ and a cut set of ECGS with M_ECGS_. We first consider the case that we are given one *specific* MCS of ECC2 (denoted by M^*^_ECC2_) and classify each single M_ECGS_ with respect to M^*^_ECC2_ (Fig. 3). Obviously, no MCS of ECGS can be a proper subset of any MCS in ECC2. Hence, every M_ECGS_ is either a superset (class A in Fig. 3) or not a superset (class B) of M^*^_ECC2_. In turn, if an M_ECGS_ is a superset of M^*^_ECC2_ then it belongs to one of four possible subclasses (A.1-A.4 in Fig. 3). In rare cases, an M_ECGS_ is identical to M^*^_ECC2_ (subclass A.1), hence, the knockouts of M^*^_ECC2_ are also sufficient in ECGS to obtain the desired phenotype. An MCS of ECGS belongs to subclass A.2 if it contains all reactions of M^*^_ECC2_ complemented by further targets exclusively contained in the ECGS (and not in the ECC2) network to block additional unfavorable pathways in ECGS. Importantly, if the given M^*^_ECC2_ is not a valid MCS in ECGS (no M_ECGS_ of type A.1 exists for M^*^_ECC2_), then some M_ECGS_ of type A.2 *must* exist: knocking out the targets from M^*^_ECC2_ and all reactions from ECGS that are not contained in ECC2 (as they were deleted during the reduction process) will lead to a solution space in ECGS that is identical to the one obtained when deleting only M^*^_ECC2_ in ECC2. Clearly, not all the reactions exclusively contained in ECGS need normally be deleted to block flux distributions feasible in ECGS but not in ECC2; a *minimal set* of reaction deletions will be sufficient which is then combined with M^*^_ECC2_. We can thus conclude that any MCS of ECCS, that is not a cut set in ECGS, can *always* be extended to a valid MCS of type A.2 in ECGS. Hence, for a given M^*^_ECC2_, there is always either one M_ECGS_ of type A.1 or one or several M_ECGS_ of type A.2. The other two possible subclasses A.3 and A.4 arise if M^*^_ECC2_ is complemented by deletions of at least one additional reaction from ECC2 either without (A.3 in Fig. 3) or with (A.4) additional deletions of reactions contained in ECGS but not in ECC2. In contrast to A.1/A.2, existence of M_ECGS_ of type A.3 and A.4 is not guaranteed.

Cut sets in ECGS which are not supersets of the given M^*^_ECC2_ (class B in Fig. 3) *must* involve some reaction knockouts within ECC2 since otherwise undesired flux distributions feasible in the subnetwork ECC2 would not be blocked within ECGS. It also follows that the set of deleted reactions in the ECC2 subnetwork cannot be a subset of *any* cut set in ECC2. Two subcases of type B can be distinguished: the knockouts made in the ECC2 subnetwork may (B.2) or may not (B.1) be combined with deletions of reactions exclusively contained in the ECGS network.

So far we discussed the relationships between one *given* cut set M^#^_ECC2_ in ECC2 with respect to each cut set in ECGS. Every cut set of ECGS belongs then to exactly one subclass. Notably, for different cut sets from ECC2, a certain cut set M^#^_ECGS_ can be of class A or B. If it is of type A.1 or A.2 for one particular M^*^_ECC2,_ then it cannot be of class A for any other MCS of ECC2 (hence, it will be of type B for all other cut sets in ECC2). If it is of type A.3 or A.4 for M^*^_ECC2_ then it can be of type A.3 or A.4 for other MCS of ECC2 (but it cannot change from A.3 to A.4 or vice versa). Of special interest are MCS of ECGS that are exclusively of type B for all MCS of ECC2 and thus not a superset (extension) of any M^*^_ECC2_. Those cut sets of ECGS involve deletion patterns in the ECC2 part that were not allowed in cut sets of ECC2 because they would violate some of the specified desired phenotypes, for example, if some of the reactions knocked-out would be essential for growth or product synthesis in ECC2 (but not in ECGS). For this reason, such MCS in ECGS may, in the extreme case, comprise fewer interventions than the smallest MCS in ECC2 (such a “super small” MCS did not exist in ECGS in our case study). All relationships between cut sets discussed above hold true not only for ECGS and ECC2 but for any pair of a (full) metabolic network and a subnetwork of it.

We analyzed the relationships between the calculated MCS in ECC2 and ECGS for the ethanol case study (Table 5) with respect to the above introduced classification scheme. 1,082 of the 44,066 MCS in ECGS establish relationships of class A since they are supersets of some cut sets in ECC2. No MCS of ECC2 was a valid MCS in network ECGS, hence, no cut set of type A.1 exists. Further, no MCS of type A.2 was found, indicating that the smallest MCS of this type (which *must* exist in ECGS) will have at least 11 reaction deletions. 12 MCS of ECGS are of type A.3 and thus extensions of MCS from ECC2 involving additional reaction deletions only in the ECC2 part. Finally, 1,070 MCS of ECGS establish relationships of type A.4. All found 1,082 MCS in ECGS of class A are supersets of 18 distinct MCS of ECC2. For each of the 4 MCS in ECC2 with cardinality 5 there exist MCS in network ECGS with 9 reaction deletions which include all deletions of the respective MCS of network ECC2 and additional deletions. Further, for 14 MCS in ECC2 with cardinality 6 there exist superset MCS in ECGS with two to four additional reaction deletions. For the remaining 2,154 MCS of ECC2, no supersets have been found among the calculated MCS in network ECGS. Since, as explained above, MCS of type A.1 or A.2 *must* exist, this indicates that the corresponding MCS in ECGS have cardinalities above ten. Lastly, 44,066 − 1,082 = 42,984 of the MCS of cardinality up to ten in ECGS are not a superset of any MCS in ECC2, hence, are exclusively of type B.1 (221) or B.2 (42763).

### MCS of the core model can be used to derive genome-scale MCS

The fact that any MCS found in the reduced network ECC2 will be a subset of one or several corresponding MCS in the full network ECGS can be used to construct MCS for the genome-scale model. The strategy is to augment a “seed” MCS from ECC2 by additional knockouts to obtain valid MCS for ECGS. Although all cut sets of type A.2, A.3, and A.4 (Fig. 3) could, in principle, be generated by the algorithm described below, we will focus on cut sets of type A.2, where only deletion of reactions contained in ECGS but not in ECC2 are added to the seed. As already pointed out, cut sets of type A.2 will always exist in ECGS and could be generated in two different ways. In a *top-down* approach, we could initially combine the knockouts of the seed MCS with knockouts of *all* reactions from ECGS that are not contained in ECC2. As already discussed, this would result in a valid but, most likely, non-minimal cut set. A constrained *minimal* cut set for ECGS can afterwards be found by iteratively removing knockout candidates from this cut set and validating *a posteriori* if the undesired phenotype is still blocked. If the removal of a reaction from the cut set violates the target constraints this reaction is reinserted and the cut set is eventually identified as a valid MCS if no further reaction can be removed.

A “bottom-up” or “expansion” approach would tackle the problem of finding smallest MCS in a more directed manner: Again, a MCS of ECC2 is used as a *seed* of reaction knockouts for ECGS and the corresponding reactions are inactivated in the ECGS model. Subsequently, all MCS in this model are computed but allowing knockouts only in reactions contained in the ECGS but not in the ECC2 model. By excluding all reactions from ECC2 as knockout candidates for ECGS, the remaining search space is considerably reduced (here by 499 reactions). The union of the seed MCS together with the MCS found in the ECGS network yields a valid MCS for the full ECGS network. In principle, this procedure can be used to determine all MCS in ECGS of type A.2 (and MCS of type A.1 can be easily identified beforehand).

We pursued the expansion approach to identify additional knockout strategies for ECGS based on the MCS from ECC2. Each of the 2176 MCS of ECC2 was used as seed in ECGS and corresponding MCS for ECGS with up to 15 additional reaction deletions were identified. Since the (up to 10) reactions of the respective seed MCS from ECC2 are inactivated prior to the computation of corresponding superset MCS in ECGS, the computed MCS can contain up to 25 interventions. The results of these calculations can be summarized as follows. For 1020 (of the 2176) seed MCS from ECC2 we found at least one MCS in ECGS with maximal 15 additional reaction deletions. For 1156 seeds no MCS could be identified in ECGS indicating that the allowed 15 reaction deletions do still not suffice to obtain valid MCS for network ECGS. If the MCS of ECC2 with cardinality 5 are used as seeds, at least 8 additional knockouts (of reactions only contained in ECGS) were necessary to achieve a valid MCS in ECGS (with overall cardinality of 13). However, the smallest MCS results from seeds of cardinality 6 where it is sufficient to delete 5 additional reactions resulting in valid MCS for network ECGS with cardinality 11. Recall here that we predicted before that the smallest MCS of type A.2 would need at least 11 reaction deletions as no MCS of this type A.2 exist up to cardinality 10. The biggest MCS found in this way for network ECGS had as much as 23 reaction deletions: 8 from the seeds and 15 additional knockouts. Again, all additional knockouts were restricted to ECGS reactions not contained in ECC2.

We exemplarily discuss specific knockout strategies that have been identified by the different approaches. A specific MCS identified in the reduced network ECC2 suggests deletion of the six reactions i) acetate kinase (R_ACK), ii) phosphogluconate dehydrogenase (R_GND), iii) hexokinase (R_HEX1), iv) D-lactate dehydrogenase (R_LDH_D), v) pyruvate formate lyase (R_PFL), and vi) glucose-6-phosphate isomerase (R_PGI) (we denote this MCS by M1). This knockout set is a valid MCS in ECC2 but does not yet enforce the desired phenotypic behavior (coupling of growth with ethanol synthesis) in network ECGS. However, by enumerating MCS up to size ten in ECGS we found a valid MCS of ECGS that extends M1 with two further reaction deletions, namely acetaldehyde dehydrogenase (R_ACALD) and either xylose isomerase (R_XYLI2) or hexokinase (HEX7). Reaction R_ACALD is contained in network ECC2 whereas reactions R_XYLI2 and R_HEX7 are only part of ECGS. These two MCS are thus of type A.4 and cannot be found with the described expansion approach starting with M1 as a seed in ECGS. However, if we used the above MCS M1 from ECC2 as a seed in ECGS and allow only reactions from ECGS that are not contained in network ECC2 as knockout candidates, at least six additional reaction knockouts are necessary to obtain a valid MCS. For example, additional deletions of R_XYLI2 or HEX7 together with NAD dependent aldehyde dehydrogenase (R_ALDD2x), NADP dependent aldehyde dehydrogenase (R_ALDD2y), citrate lyase (R_CITL), gluconokinase (R_GNK) and acetyl-CoA:hexanoate-CoA transferase (R_HXCT) result in a valid MCS for ECGS and is found by our procedure. These knockout strategies reveal that the deletion of R_ACALD (in the ECC2 subnetwork) is equivalent to the knockout of R_ALDD2x, R_ALDD2y, R_CITL, R_GNK and R_HXCT in the ECGS network.

Generally, elimination of the R_ACALD reaction (catalyzed by the acetaldehyde dehydrogenase (*adhE*)) was identified as a knockout candidate in several MCS in ECGS but never in ECC2 (where it is the only pathway to produce ethanol). Some alternative ethanol synthesis pathways must therefore exist in ECGS that, with the suggested knockout strategies, would lead to a high ethanol yield. In fact, in ECGS, acetaldehyde can be derived from acetyl-CoA and glycine that are condensed to L-2-Amino-3-oxobutonate (R_GLYAT) which is then reduced to L-allo-threonine via reaction R_ATHRDHr and finally split to acetaldehyde and glycine by R_THRA2i. However, since the acetaldehyde dehydrogenase (*adhE*) is part of the main route for ethanol production in *E. coli* and since *adhE* knockout mutants are known to show no relevant ethanol excretion at all^32^,^33^ a beneficial effect of those engineering strategies appears questionable (though it cannot be excluded and may deliver non-intuitive results).

To summarize, our case study showed that metabolic intervention strategies can be exhaustively enumerated in the reduced network ECC2. The MCS found in the ECC2 network for enhancing ethanol synthesis show significant overlap with the smallest MCS identifiable in ECGS. Discrepancies mainly arise due to (a) additional pathways in ECGS having a zero or low ethanol yield (which must be deleted in ECGS) and (b) additional high-yield ethanol synthesis pathways (which give rise to new intervention strategies in ECGS). For a product of the central metabolism such as ethanol, we expect that a large number of MCS found in ECC2 are sufficient to reach the desired production phenotype. In fact, successful *E. coli* strains for ethanol overproduction reported in the literature contained only deletions in the central metabolism^34^,^35^,^36^ and the example of the suggested deletion of the acetaldehyde dehydrogenase reaction R_ACALD in the ECGS model shows that “exotic” intervention strategies (that redirect the flux to alternative product synthesis pathways) might be found in the genome-scale model whose relevance is unclear but cannot be excluded *a priori*. Generally, if calculated (smallest) MCS in ECGS require a huge and unrealistic number of knockouts or if a calculation of (large sets of) MCS for a given intervention problem is not feasible at all in the ECGS model, it would thus be practical to start with a MCS calculated in the core network ECC2. If experimental implementation of all knockouts contained in the MCS of ECC2 is not sufficient, one may use our expansion algorithm to expand the seed MCS from the ECC2 model to obtain a valid MCS for the ECGS model (whose existence is guaranteed).

## Discussion

The central goal of this contribution was the development of a reference metabolic core model of the central metabolism of *E. coli* that is stoichiometrically consistent to and maintains several key properties of the most recent and well-curated genome-scale model of this organism (*i*JO1366). In addition, for the first time, we systematically compared key properties of the derived metabolic core model and of its genome-scale parent model.

For deriving the core model we used the recently introduced *NetworkReducer* algorithm together with a list of protected parts and phenotypes. This list was carefully compiled to ensure that the core model reflects the degrees of freedom in the central metabolism of *E. coli* while it was allowed to neglect redundancy along biosynthetic routes. The derived *EColiCore2* network is a subnetwork of *i*JO1366 still comprising 499 reactions and 486 internal metabolites. However, ECC2 is small enough for detailed studies that would not be possible in the genome-scale model. In particular, all elementary modes for growth on the included substrates can be calculated on a standard personal computer in reasonable time. In addition, by compressing (mainly biosynthetic) linear reaction chains to condensed overall reactions a compressed network version of ECC2, ECC2comp, was derived which contains 82 reactions and 54 internal metabolites. The solution space of ECC2comp is equivalent to ECC2 since every steady state flux distribution in ECC2comp can be uniquely mapped to one of ECC2 and vice versa. ECC2comp might be more practical and efficient for some applications. For example, less working memory is required for sophisticated calculations. Further, the reduced number of reactions and metabolites allows for a clear and concise graphical representation of the whole network (Fig. 1). On the other hand, the compressed version does not explicitly represent the pathways for synthesizing biomass components and uses a modified stoichiometry for the biomass synthesis reaction. The latter, however, is consistent with the original stoichiometry and balances the drain of carbon from precursors contained in the compressed model.

We performed a comprehensive analysis and comparison of different properties of ECC2 and its genome-scale parent model ECGS to demonstrate that ECC2 is a reasonable reduction of ECGS. ECC2 preserves key phenotypic behaviors and properties and allows for the analysis of major metabolic fluxes and the elucidation of engineering strategies in the central metabolism of *E. coli* that would be difficult to obtain or interpret with ECGS due to its tremendous complexity. We also characterized major differences between both models and showed that these are indeed mainly related to redundancies in anabolic pathways in ECGS. For example, comparing reaction essentialities reveals that many reactions were predicted to be essential in ECC2 that where not essential in ECGS. This is almost exclusively the case for reactions of the anabolism needed to synthesize precursors for biomass synthesis. During the reduction process many parallel reactions have been deleted that could alternatively synthesize these precursors. However, reaction essentialities for the reactions of the central metabolism in ECC2 are in almost perfect concordance with ECGS. A comparison of production envelopes and producibilities of metabolites in ECC2 and ECGS showed also a good agreement. Some deviations, especially related to acetate and acetyl-CoA metabolism, presumably arise due to some overoptimistic predictions in ECGS. Furthermore, for a few reactions of the central metabolism, we found larger differences in the flux ranges that mainly arise due to the activity of certain pathways or cycles in ECGS many of which are thermodynamically infeasible or at least unlikely (due to extremely large fluxes). These examples demonstrate the trade-off in analyzing a core vs. a full genome-scale network: unrealistic or overoptimistic behaviors represented in the genome-scale model (e.g., due to unknown flux capacities) can be excluded in a core model, however, unknown and non-intuitive pathways of possibly higher relevance might be missed when focusing only on the well-examined part of the metabolism.

We also studied the value of the core model for calculating relevant metabolic engineering strategies by means of a case study with ethanol as product of interest. As expected, cut sets found in ECC2 for overproduction of ethanol are usually not sufficient to completely block all potentially undesired phenotypes (with low product yield) in ECGS but already indicate key targets (and combinations thereof) which are also relevant for ECGS. Analysis of the smallest cut sets in ECGS showed that non-intuitive intervention strategies may exist in the genome-scale model which either target additional unfavorable routes that were not contained in the ECC2 model or/and redirect carbon flux to hidden (unknown) product synthesis pathways in the full network. While the cut sets of the ECGS model are complete and ensure that the desired phenotypes are really achieved in the full model, the relevance of some of the found alternative targets (with the acetaldehyde dehydrogenase as a prominent example) and pathways is questionable. Generally, one can expect that intervention strategies calculated in ECC2 for products of the central metabolism (e.g., standard fermentation products) will have a better predictivity (most relevant pathways for their production are contained in the model) than those calculated for products of the anabolism where relevant - favorable and unfavorable - pathways might have been removed in the pruning step.

We further clarified the relationships between cuts sets of the full and of a subnetwork. With respect to a given subnetwork (here ECC2) one can distinguish two major classes of cut sets in the full (ECGS) model: a cut set in ECGS is either a superset of one (or several) cut sets found in ECC2 (which also includes the possible but rare case that a cut set in ECC2 is also a valid cut set in ECGS), or it is not a superset of any cut set in ECC2 and therefore involves other deletion patterns in the ECC2 subnetwork possibly combined with further knockouts in reactions exclusively contained in ECGS. Whereas cut sets of the latter type can therefore not be obtained or deduced from ECC2, we demonstrated that cut sets in ECGS that are supersets of cut sets from ECC2 can always be obtained by expanding seed cut sets in ECC2. Thus, ECC2 can initially be used to find intervention strategies leading to desired phenotypes in the core model. If a particular cut set was experimentally not successful (the mutant shows still an unfavorable phenotype in the experiment) or if one is interested to determine (some or more) full intervention strategies in the ECGS model, the respective knockout strategy of ECC2 can be used as a seed and then be extended by further knockouts to a valid MCS for ECGS. We demonstrated that this approach can be used to determine thousands of additional valid MCS for ECGS with higher cardinalities which could not be calculated before.

A major advantage of ECC2 is that it is a strict submodel of its genome-scale parent model ECGS so that results from ECC2 can be directly related to ECGS. In fact, this property was used to determine cut sets in the ECGS model from seed cut sets calculated in ECC2. Furthermore, all flux distributions in ECC2 are valid solutions in ECGS and, likewise, all EM of ECC2 are equally valid in ECGS. Hence, analyzing ECC2 means analyzing a subset of the feasible phenotypes of ECGS and ECC2 was derived in such a way that metabolic fluxes in the central metabolism of *E. coli* are well reflected in the reduced model.

As desired, ECC2 allows for the full enumeration of EM and metabolic engineering strategies and it is readily usable for educational purposes due to its appropriate scope, size, and clarity. If certain pathways or/and metabolites contained in ECGS but not in the ECC2 model become of interest in a particular application, they might be added to the core model which should still keep the model size low enough to perform detailed calculations (e.g. EM enumeration). Likewise, if one wants to protect other phenotypes from the genome-scale model, the reduction process can be repeated with adapted constraints.

With the results presented in this study, we believe that ECC2 holds the potential to become a reference metabolic model for the central metabolism of *E. coli*, similar to its well-curated and evolved genome-scale parent model *i*JO1366.

## Additional Information

**How to cite this article**: Hädicke, O. and Klamt, S. *EColiCore2*: a reference network model of the central metabolism of *Escherichia coli* and relationships to its genome-scale parent model. *Sci. Rep.* **7**, 39647; doi: 10.1038/srep39647 (2017).

**Publisher's note:** Springer Nature remains neutral with regard to jurisdictional claims in published maps and institutional affiliations.

## Supplementary Material

Supplementary Information

Supplementary Dataset 1

Supplementary Dataset 2

Supplementary Dataset 3

## Figures and Tables

**Table 1:** Protected phenotypes (maximal growth rate and production rates) in ECGS used to derive the core model ECC2.

Criteria	Description	ECGS	Desired	Reached (ECC2)
C1	μ_max_ for aerobic growth on glucose	0.982	0.962	0.982 (>99.9%)
C2	μ_max_ for anaerobic growth on glucose	0.289	0.283	0.289 (>99.9%)
C3	μ_max_ for aerobic growth on acetate	0.247	0.242	0.244 (98.8%)
C4	μ_max_ for aerobic growth on succinate	0.493	0.483	0.492 (>99.9%)
C5	μ_max_ for aerobic growth on glycerol	0.563	0.552	0.563 (>99.9%)
C6	Max. rate of anaerobic formate production	49.4	>0.1	24.0 (48.6%)
C7	Max. rate of anaerobic lactate production	20.0	>0.1	20.0 (100.0%)
C8	Max. rate of anaerobic succinate production	16.9	>0.1	16.9 (100.0%)
C9	Max. rate of anaerobic hydrogen production	101.1	>0.1	24.0 (23.7%)
C10	Max. rate of anaerobic ethanol production	20.0	>0.1	20.0 (100.0%)
C11	Max. rate of anaerobic acetate production	27.5	>0.1	10.0 (36.4%)

ATP maintenance flux was fixed to 3.15 mmol/gDW/h, maximal substrate uptake fluxes to 10 mmol/gDW/h and oxygen uptake flux to zero for anaerobic conditions. Growth rate (C1-C5) is given in h^−1^ and production rates (C6-C11) refer to mmol/gDW/h glucose.

**Table 2:** Properties of the genome-scale (ECGS), the core (ECC2) and the compressed core (ECC2comp) *E. coli* network model discussed in the text.

	*i*JO1366	ECGS	ECC2	ECC2comp
# reactions (reversible)	2583 (635)	2583 (636)	499 (198)	82 (32) [79 (35)]
# internal metabolites	1805	1807	486	54 [61]
# external metabolites	0	331	33	40 [33]
# degrees of freedom	817	815	25	28 [25]
# conservation relations	39	39	12	0 [7]
# subsets of coupled reactions (# containing reactions)	487 (1456)	330 (968)	25 (462)	15 (32)

ECGS is a slightly modified version of *i*JO1366 (see text). The number of reversible reactions for *i*JO1366 and ECGS refers to the used minimal medium configuration. The numbers in the brackets in the ECC2comp column refer to the compressed model before splitting three reversible exchange reactions and removing conservation relations by setting one metabolite per conservation relation to external (see text). ECGS, ECC2 and ECC2comp are available as SBML files in the Supplementary Material and as corresponding *CellNetAnalyzer* projects at http://www2.mpi-magdeburg.mpg.de/projects/cna/cna.html.

**Table 3:** Comparison of flux ranges, reaction essentialities and metabolite producibilites in ECC2 and ECGS for growth on glucose.

Property	Proportions
*Flux ranges (under growth-optimality; F*_*i*_ *is the difference of maximal and minimal flux of reaction i)*	
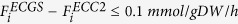	290/499 (58.1%)
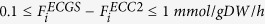	153/499 (30.7%)
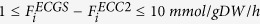 |	29/499 (5.8%)
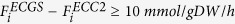	27/499 (5.4%)
*Essentialities of reactions*	
Essential for growth (ECC2/ECGS)	411/290
● for protected reactions (ECC2/ECGS)	7/6
Not essential for growth but growth-rate limiting (ECC2/ECGS)	17/16
● for protected reactions (ECC2/ECGS)	8/7
No effect, growth rate >95% of μ_max_ (ECC2/ECGS)	71/193
● for protected reactions (ECC2/ECGS)	50/52
*Producibilities of metabolites*	
Maximal product yield in ECC2 >95% of maximal product yield in ECGS	378/486 (77.8%)
Maximal product yield in ECC2 >80% and <95% of maximal product yield in ECGS	55/486 (11.3%)
Maximal product yield in ECC2 <80% of maximal product yield in ECGS	53/486 (10.9%)

Comparisons were restricted to those reactions and internal metabolites that are present in ECC2.

**Table 4:** Number of elementary modes for the four different substrates in ECC2 (and ECC2comp).

	Growth on glucose	Growth on acetate	Growth on succinate	Growth on gycerol	Anaerobic growth on glucose
Total number of EM	3,263,223(2,906,916)	4,210(2,417)	46,758(36,342)	1,282,520(1,224,524)	144,257(135,571)
(with growth)					

**Table 5:** Number of minimal cut sets for the ethanol case study in networks ECC2 and ECGS.

Cardinality	ECC2	ECGS
1	0	0
2	0	0
3	0	0
4	0	0
5	4	0
6	40	123
7	700	626
8	736	2627
9	316	10472
10	380	30218
Total	2176	44066
