# Corrigendum: *EColiCore2*: a reference network model of the central metabolism of *Escherichia* *coli* and relationships to its genome-scale parent model

**DOI:** 10.1038/srep44520

**Published:** 2017-03-16

**Authors:** Oliver Hädicke, Steffen Klamt

Scientific Reports 7: Article number: 3964710.1038/srep39647; published online: 01 03 2017; updated: 03 16 2017

The SBML models provided in the original Supplementary Information files published with this Article contained formatting errors. These errors have been corrected in the Supplementary Information files that now accompany the Article.

