# Supplementary material for: EColiCore2: a reference network model of the central metabolism of Escherichia coli and relationships to its genome-scale parent model
Source: Sci Rep. 2017 Jan 3;7:39647. doi: 10.1038/srep39647 (PMC5206746; doi:10.1038/srep39647)
Supplement: Supplementary Information [file srep39647-s1.pdf]

Supplementary Information

EColiCore2: a reference network model of the central metabolism of *Escherichia coli* and relationships to its genome-scale parent model

Oliver Hädicke and Steffen Klamt*

Max Planck Institute for Dynamics of Complex Technical Systems

Sandtorstrasse 1, 39106 Magdeburg, Germany

* To whom correspondence should be addressed:

Steffen Klamt

Max Planck Institute for Dynamics of Complex Technical Systems, Germany

Sandtorstrasse 1

D-39106 Magdeburg, Germany

Phone: ++49 391 6110 480

Email: klamt@mpi-magdeburg.mpg.de

1. Protected reactions and metabolites of *i*JO1366 used in the reduction process

Reaction and metabolite names as in Orth et al.[1].

#	Reaction ID	Stoichiometry
		Glycolysis / Gluconeogenesis
1	R_PGI	M_g6p_c <==> M_f6p_c
2	R_PFK	M_atp_c + M_f6p_c ==> M_adp_c + M_fdp_c + M_h_c
3	R_FBP	M_fdp_c + M_h2o_c ==> M_f6p_c + M_pi_c
4	R_FBA	M_fdp_c <==> M_dhap_c + M_g3p_c
5	R_TPI	M_dhap_c <==> M_g3p_c
6	R_GAPD	M_g3p_c + M_nad_c + M_pi_c <==> M_13dpg_c + M_h_c + M_nadh_c
7	R_PGK	M_3pg_c + M_atp_c <==> M_13dpg_c + M_adp_c
8	R_PGM	M_2pg_c <==> M_3pg_c
9	R_ENO	M_2pg_c <==> M_h2o_c + M_pep_c
10	R_PYK	M_adp_c + M_h_c + M_pep_c ==> M_atp_c + M_pyr_c
11	R_PPS	M_atp_c + M_h2o_c + M_pyr_c ==> M_amp_c + 2 M_h_c + M_pep_c + M_pi_c
		Glycerol metabolism
12	R_GLYK	M_atp_c + M_glyc_c ==> M_adp_c + M_glyc3p_c + M_h_c
13	R_G3PD2	M_glyc3p_c + M_nadp_c <==> M_dhap_c + M_h_c + M_nadph_c
14	R_GLYCDx	M_glyc_c + M_nad_c ==> M_dha_c + M_h_c + M_nadh_c
15	R_G3PD5	M_glyc3p_c+M_q8_c==>M_dhap_c+M_q8h2_c
16	R_F6PA	M_f6p_c<==>M_dha_c+M_g3p_c
		Pentose Phosphate Pathway
17	R_G6PDH2r	M_g6p_c + M_nadp_c <==> M_6pgl_c + M_h_c + M_nadph_c
18	R_PGL	M_6pgl_c + M_h2o_c ==> M_6pgc_c + M_h_c
19	R_GND	M_6pgc_c + M_nadp_c ==> M_co2_c + M_nadph_c + M_ru5p_DASH_D_c
20	R_RPE	M_ru5p_DASH_D_c <==> M_xu5p_DASH_D_c
21	R_RPI	M_r5p_c <==> M_ru5p_DASH_D_c
22	R_TKT1	M_r5p_c + M_xu5p_DASH_D_c <==> M_g3p_c + M_s7p_c
23	R_TALA	M_g3p_c + M_s7p_c <==> M_e4p_c + M_f6p_c
24	R_TKT2	M_e4p_c + M_xu5p_DASH_D_c <==> M_f6p_c + M_g3p_c
		Entner Doudoroff Pathway
25	R_EDA	M_2ddg6p_c ==> M_g3p_c + M_pyr_c
26	R_EDD	M_6pgc_c ==> M_2ddg6p_c + M_h2o_c
		Glyoxylate shunt
27	R_ICL	M_icit_c ==> M_glx_c + M_succ_c
28	R_MALS	M_accoa_c + M_glx_c + M_h2o_c ==> M_coa_c + M_h_c + M_mal_DASH_L_c
		TCA-cycle
29	R_PDH	M_coa_c + M_nad_c + M_pyr_c ==> M_accoa_c + M_co2_c + M_nadh_c
30	R_CS	M_accoa_c + M_h2o_c + M_oaa_c ==> M_cit_c + M_coa_c + M_h_c
31	R_ACONTa	M_cit_c <==> M_acon_DASH_C_c + M_h2o_c

32	R_ICDHyr	M_icit_c + M_nadp_c <==> M_akg_c + M_co2_c + M_nadph_c
33	R_AKGDH	M_akg_c + M_coa_c + M_nad_c ==> M_co2_c + M_nadh_c + M_succoa_c
34	R_SUCOAS	M_atp_c + M_coa_c + M_succ_c <==> M_adp_c + M_pi_c + M_succoa_c
35	R_SUCDi	M_q8_c + M_succ_c ==> M_fum_c + M_q8h2_c
36	R_FRD2	M_fum_c + M_mql8_c ==> M_mqn8_c + M_succ_c
37	R_FUM	M_fum_c + M_h2o_c <==> M_mal_DASH_L_c
38	R_MDH	M_mal_DASH_L_c + M_nad_c <==> M_h_c + M_nadh_c + M_oaa_c
39	R_ACONTb	M_acon_DASH_C_c+M_h2o_c<==>M_icit_c
		Anaplerotic reactions
40	R_ME2	M_mal_DASH_L_c + M_nadp_c ==> M_co2_c + M_nadph_c + M_pyr_c
41	R_ME1	M_mal_DASH_L_c + M_nad_c ==> M_co2_c + M_nadh_c + M_pyr_c
42	R_PPCK	M_atp_c + M_oaa_c ==> M_adp_c + M_co2_c + M_pep_c
43	R_PPC	M_co2_c + M_h2o_c + M_pep_c ==> M_h_c + M_oaa_c + M_pi_c
		Fermentative reactions
44	R_ACALD	M_acald_c + M_coa_c + M_nad_c <==> M_accoa_c + M_h_c + M_nadh_c
45	R_ALCD2x	M_etoh_c+M_nad_c<==>M_acald_c+M_h_c+M_nadh_c
46	R_PTAr	M_accoa_c+M_pi_c<==>M_actp_c+M_coa_c
47	R_ACKr	M_ac_c+M_atp_c<==>M_actp_c+M_adp_c
48	R_PFL	M_coa_c+M_pyr_c==>M_accoa_c+M_for_c
49	R_LDH_D	M_lac_DASH_D_c+M_nad_c<==>M_h_c+M_nadh_c+M_pyr_c
50	R_POX	M_h2o_c+M_pyr_c+M_q8_c==>M_ac_c+M_co2_c+M_q8h2_c
51	R_FHL	M_for_c+M_h_c==>M_co2_c+M_h2_c
		Methylglyoxal pathway
52	R_MGSA	M_dhap_c==>M_mthgxl_c+M_pi_c
53	R_GLYOX3	M_h2o_c+M_mthgxl_c==>M_h_c+M_lac_DASH_D_c
		Electron transport chain
54	R_NADH16pp	4M_h_c+M_nadh_c+M_q8_c<==>M_nad_c+M_q8h2_c+3M_h_p
55	R_NADH17pp	4M_h_c+M_mqn8_c+M_nadh_c==>M_mql8_c+M_nad_c+3M_h_p
56	R_CYTBO3_4pp	4M_h_c+0.5M_o2_c+M_q8h2_c==>M_h2o_c+M_q8_c+4M_h_p
57	R_NADTRHD	M_nad_c+M_nadph_c==>M_nadh_c+M_nadp_c
58	R_THD2pp	M_nadh_c+M_nadp_c+2M_h_p==>2M_h_c+M_nad_c+M_nadph_c
		Energy metabolism
59	R_ATPS4rpp	M_adp_c+M_pi_c+4M_h_p<==>M_atp_c+3M_h_c+M_h2o_c
60	R_ATPM	M_atp_c+M_h2o_c==>M_adp_c+M_h_c+M_pi_c
61	R_ADK1	M_amp_c+M_atp_c<==>2M_adp_c
		Glucose uptake
62	R_GLCptspp	M_pep_c+M_glc_DASH_D_p==>M_g6p_c+M_pyr_c
63	R_GLCt2pp	M_glc_DASH_D_p+M_h_p==>M_glc_DASH_D_c+M_h_c
64	R_HEX1	M_atp_c+M_glc_DASH_D_c==>M_adp_c+M_g6p_c+M_h_c
		Growth
65	R_Ec_biomass_iJO1366_core_53p95M	0.000223 M_10fthf_c + 2.6e-05 M_2fe2s_c + 0.000223 M_2ohph_c + 0.00026 M_4fe4s_c + 0.51369 M_ala_DASH_L_c + 0.000223

	M_amet_c + 0.29579 M_arg_DASH_L_c + 0.24105 M_asn_DASH_L_c + 0.24105 M_asp_DASH_L_c + 54.1248 M_atp_c + 0.000122 M_bmocogdp_c + 2e-06 M_btn_c + 0.005205 M_ca2_c + 0.005205 M_cl_c + 0.000576 M_coa_c + 2.5e-05 M_cobalt2_c + 0.13351 M_ctp_c + 0.000709 M_cu2_c + 0.09158 M_cys_DASH_L_c + 0.026166 M_datp_c + 0.027017 M_dctp_c + 0.027017 M_dgtp_c + 0.026166 M_dttp_c + 0.000223 M_fad_c + 0.006715 M_fe2_c + 0.007808 M_fe3_c + 0.26316 M_gln_DASH_L_c + 0.26316 M_glu_DASH_L_c + 0.61264 M_gly_c + 0.2151 M_gtp_c + 48.6015 M_h2o_c + 0.094738 M_his_DASH_L_c + 0.29053 M_ile_DASH_L_c + 0.19519 M_k_c + 0.45053 M_leu_DASH_L_c + 0.34316 M_lys_DASH_L_c + 0.15369 M_met_DASH_L_c + 0.008675 M_mg2_c + 0.000223 M_mlthf_c + 0.000691 M_mn2_c + 7e-06 M_mobd_c + 0.001831 M_nad_c + 0.000447 M_nadp_c + 0.013013 M_nh4_c + 0.000323 M_ni2_c + 0.017868 M_pe160_c + 0.054154 M_pe161_c + 0.18527 M_phe_DASH_L_c + 0.000223 M_pheme_c + 0.22106 M_pro_DASH_L_c + 0.000223 M_pydx5p_c + 0.000223 M_ribflv_c + 0.21579 M_ser_DASH_L_c + 0.000223 M_sheme_c + 0.004338 M_so4_c + 0.000223 M_thf_c + 0.000223 M_thmpp_c + 0.25369 M_thr_DASH_L_c + 0.056843 M_trp_DASH_L_c + 0.1379 M_tyr_DASH_L_c + 5.5e-05 M_udcpdp_c + 0.1441 M_utp_c + 0.42316 M_val_DASH_L_c + 0.000341 M_zn2_c + 0.019456 M_kdo2lipid4_e + 0.013894 M_murein5px4p_p + 0.045946 M_pe160_p + 0.02106 M_pe161_p ==> 53.95 M_adp_c + 53.95 M_h_c + 53.9457 M_pi_c + 0.7739 M_ppi_c + 1 gDW

2. Protected phenotypes used in the reduction process

Global constraints used for all protected phenotypes

R_EX_glc_LPAREN_e_RPAREN_ ≥ -10;
R_EX_ac_LPAREN_e_RPAREN_ ≥ -10;
R_EX_succ_LPAREN_e_RPAREN_ ≥ -10;
R_EX_glyc_LPAREN_e_RPAREN_ ≥ -10;
R_ATPM ≥ 3.15;

Constraints for the protected phenotypes C1 – C11 (see Table 1 in main document)

C1 – aerobic growth on glucose

R_Ec_biomass_iJO1366_core_53p95M ≥ 0.962;
R_EX_ac_LPAREN_e_RPAREN_ ≥ 0;
R_EX_succ_LPAREN_e_RPAREN_ ≥ 0;
R_EX_glyc_LPAREN_e_RPAREN_ ≥ 0;

The following reactions were set to inactive to avoid essentiality of these reactions under aerobic conditions for growth on glucose:

R_EX_for_LPAREN_e_RPAREN_ = R_EX_etoh_LPAREN_e_RPAREN_= R_EX_lac_DASH_D_LPAREN_e_RPAREN_= R_EX_succ_LPAREN_e_RPAREN_ = R_EX_ac_LPAREN_e_RPAREN_ = R_EX_h2_LPAREN_e_RPAREN_ = R_PFL = R_MALS = R_FRD2 = R_POR5 = R_AACTOOR = R_ASPO6 = R_GGPTRCO = R_MOX = R_MTRPOX = R_PDX5POi = R_PYAM5PO = R_SARCOX = R_SPODM = R_URIC = 0

C2 - anaerobic growth on glucose

R_Ec_biomass_iJO1366_core_53p95M ≥ 0.283;
R_EX_o2_LPAREN_e_RPAREN_ = 0;
R_EX_ac_LPAREN_e_RPAREN_ ≥ 0;
R_EX_succ_LPAREN_e_RPAREN_ ≥ 0;
R_EX_glyc_LPAREN_e_RPAREN_ ≥ 0;

C3 - aerobic growth on acetate

R_Ec_biomass_iJO1366_core_53p95M ≥ 0.242;
R_EX_glc_LPAREN_e_RPAREN_ ≥ 0;
R_EX_succ_LPAREN_e_RPAREN_ ≥ 0;
R_EX_glyc_LPAREN_e_RPAREN_ ≥ 0;

C 4 - aerobic growth on succinate

R_Ec_biomass_iJO1366_core_53p95M ≥ 0.483;
R_EX_glc_LPAREN_e_RPAREN_ ≥ 0;
R_EX_ac_LPAREN_e_RPAREN_ ≥ 0;
R_EX_glyc_LPAREN_e_RPAREN_ ≥ 0;

C5 - aerobic growth on glycerol

R_Ec_biomass_iJO1366_core_53p95M ≥ 0.552;
R_EX_glc_LPAREN_e_RPAREN_ ≥ 0;
R_EX_ac_LPAREN_e_RPAREN_ ≥ 0;
R_EX_succ_LPAREN_e_RPAREN_ ≥ 0;

C6 - Formate production (growth on glucose)

R_EX_for_LPAREN_e_RPAREN_ ≥ 0.1;
R_EX_ac_LPAREN_e_RPAREN_ ≥ 0;
R_EX_succ_LPAREN_e_RPAREN_ ≥ 0;
R_EX_glyc_LPAREN_e_RPAREN_ ≥ 0;

C7 - Lactate production (growth on glucose)

R_EX_lac_DASH_D_LPAREN_e_RPAREN_ ≥ 0.1;
R_EX_ac_LPAREN_e_RPAREN_ ≥ 0;
R_EX_succ_LPAREN_e_RPAREN_ ≥ 0;
R_EX_glyc_LPAREN_e_RPAREN_ ≥ 0;

C8 - Hydrogen production (growth on glucose)

R_EX_h2_LPAREN_e_RPAREN_≥ 0.1;
R_EX_ac_LPAREN_e_RPAREN_ ≥ 0;
R_EX_succ_LPAREN_e_RPAREN_ ≥ 0;
R_EX_glyc_LPAREN_e_RPAREN_ ≥ 0;

C9 - Succinate production (growth on glucose)

R_EX_succ_LPAREN_e_RPAREN_ ≥ 0.1;
R_EX_ac_LPAREN_e_RPAREN_ ≥ 0;
R_EX_glyc_LPAREN_e_RPAREN_ ≥ 0;

C10 – Ethanol production (growth on glucose)

R_EX_etoh_LPAREN_e_RPAREN_≥ 0.1;
R_EX_ac_LPAREN_e_RPAREN_ ≥ 0;
R_EX_succ_LPAREN_e_RPAREN_ ≥ 0;
R_EX_glyc_LPAREN_e_RPAREN_ ≥ 0;

C11 – Acetate production (growth on glucose)

R_EX_ac_LPAREN_e_RPAREN_ ≥ 0.1;
R_EX_succ_LPAREN_e_RPAREN_ ≥ 0;
R_EX_glyc_LPAREN_e_RPAREN_ ≥ 0;

References

1 Orth, J. D. *et al.* A comprehensive genome-scale reconstruction of *Escherichia coli* metabolism--2011. *Mol Syst Biol* **7**, 535.
